# CRISPR/Cas9 Targets Chicken Embryonic Somatic Cells *In Vitro* and *In Vivo* and generates Phenotypic Abnormalities

**DOI:** 10.1038/srep34524

**Published:** 2016-10-03

**Authors:** Kwaku Dad Abu-Bonsrah, Dongcheng Zhang, Donald F. Newgreen

**Affiliations:** 1 Department of Paediatrics, University of Melbourne, Parkville 3052, Australia; 2 Murdoch Childrens Research Institute, Royal Children’s Hospital, Parkville, 3052, Australia

## Abstract

Chickens are an invaluable model for studying human diseases, physiology and especially development, but have lagged in genetic applications. With the advent of Programmable Engineered Nucleases, genetic manipulation has become efficient, specific and rapid. Here, we show that the CRISPR/Cas9 system can precisely edit the chicken genome. We generated *HIRA, TYRP1, DICER, MBD3, EZH2*, and 6 other gene knockouts in two chicken cell lines using the CRISPR/Cas9 system, with no off-target effects detected. We also showed that very large deletions (>75 kb) could be achieved. We also achieved targeted modification by homology-directed repair (HDR), producing MEN2A and MEN2B mutations of the *RET* gene. We also targeted *DGCR8* in neural cells of the chicken embryo by *in vivo* electroporation. After FACS isolation of transfected cells, we observed appropriate sequence changes in *DGCR8.* Wholemount and frozen section antibody labelling showed reduction of *DGCR8* levels in transfected cells. In addition, there was reduced expression levels of *DGCR8*-associated genes *DROSHA, YPEL1* and *NGN2*. We also observed morphological differences in neural tissue and cardiac-related tissues of transfected embryos. These findings demonstrate that precisely targeted genetic manipulation of the genome using the CRISPR/Cas9 system can be extended to the highly adaptable *in vivo* chicken embryo model.

Avian embryos, chiefly the chicken (*Gallus gallus*) and quail (*Coturnix japonica*), have been a mainstay of vertebrate embryologic research for over a century^1^. The avian embryo is easy to obtain cheaply in large numbers as a result of its commercial utility. Development of avian embryos is simple to synchronise with little individual variation and benefits from excellent tables of development. Because development is external, these embryos are accessible to experimental manipulation^2^. The similarity in the general ontogeny and gene expression patterns between avian embryos and those of other vertebrates including mouse embryos indicates that Aves can serve as an excellent model for the study of genetic, molecular and biochemical mechanisms in mammals including humans. Avian models have been especially useful in following developmental pathways, for example in tracking cell movements in morphogenesis, untangling inductive pathways and deciphering differentiation pathways. The results of these have been shown to be valid for other vertebrates^3^. Avian embryos have also been instructive in pathogenesis of embryonic diseases, and also physiology, behaviour and toxicology^4^,^5^.

Avian embryology has lagged in genetic research partly because the commercial utility of poultry factored against collection of instructive mutants (as in the fly, fish and mouse) and partly because of the dearth of techniques to manipulate their genome. The introduction of *in vivo* transfection by electroporation and by viral vectors changed this dramatically, and allowed a form of conditional mutagenesis that was cheap and rapid^6^. Great ingenuity has enabled up- and down-regulation of genes to be achieved in avian embryos^7^, but these techniques largely involved adding extra genetic information in a non-targeted way, in the form of plasmids or miRNAs, either episomally, or by means that randomly integrate into the host genome^8^.

Programmable engineered nucleases (PENs) are novel technologies developed to efficiently target and alter a specific allele in the genome. These PENs, the zinc finger nucleases (ZFNs), the transcription activator-like effector nucleases (TALENs) and the clustered regular interspaced palindromic repeats (CRISPR)/Cas9 system, have been used extensively in generating and correcting mutations in cells of plants^9^, humans^10^,^11^, rodents^12^,^13^, monkeys^14^,^15^, fish^16^,^17^,^18^, fly^19^,^20^ and worm^21^ *in vitro* and *in vivo*, to generate transgenic cells, animals and plants. Recently, Park *et al*. validated the efficiency of TALENs in generating knockout chicken primordial germ cells (PGCs)^22^ and showed that TALENs can be used to efficiently modify the genomes of chickens. Here we utilise the recently described CRISPR/Cas9 system, which is a very simple but powerful tool in the editing *in vivo* of the genomes of mice^23^,^24^, in knocking-out and knocking-in of sequences in chicken cells *in vitro* and *in vivo.*

## Results

A recent report showed that the gene *Pax7* could be modified by CRISPR/Cas9 in the chicken embryo *in vivo*^25^. To explore the general applicability of this technique, we investigated a range of chicken genes on both macrochromosomes and on the unusual avian microchromosomes. The genes chosen were *DROSHA, DICER, MBD3, KIAA1279, CDKN1B, EZH2, HIRA, TYRP1, STMN2, RET* and *DGCR8* (Di George Critical Region8) (Supplementary Table 1). These genes have roles in embryonic development and the pathogenesis of embryonic diseases.

### The CRISPR/Cas9 system mediates NHEJ and HDR gene disruptions in chicken cell lines

**Figure 1:**
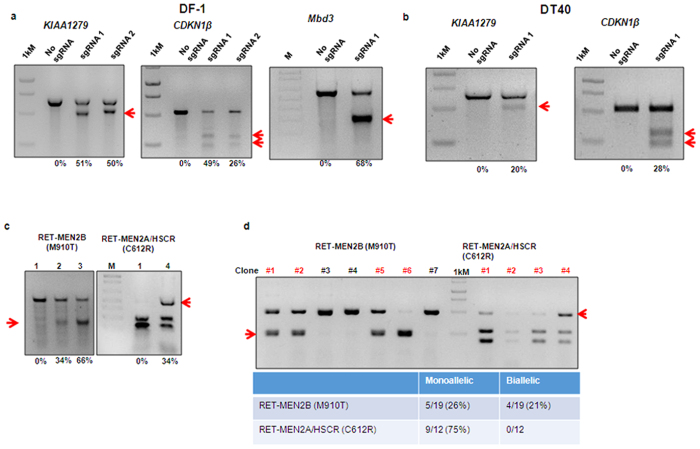
*In vitro* analysis of NHEJ and HDR genome modification (arrows) mediated by sgRNA-Cas9 system in chicken cell lines. (**a**) Frequency (%) of NHEJ mutation mediated by *KIAA1279, Cdkn1b* and *Mbd3*-targeting sgRNA-Cas9 system in chicken DF-1 cells by PCR and T7E1 assay. 1kM- 1 kbp DNA ladder, M- 100 bp DNA ladder. (**b**) Frequency (%) of NHEJ mutation mediated by *KIAA1279* and *Cdkn1b*-targeting sgRNA-CRISPR/Cas9 system in chicken lymphoma B DT40 cells by PCR and T7E1 assay. (**c**) Representative gel from DF-1 cells transfected with the RET-targeting sgRNA-Cas9 and the ssODN showing efficient integration of the HDR-based BamHI and EcoRV sequence. The frequency of HDR is represented in percentages. 1-No sgRNA, 2- MEN2B sgRNA #1 plus ssODN, 3- MEN2B sgRNA #1 and #2 plus ssODN and 4- MEN2A/HSCR sgRNA 1 plus ssODN. (**d**) Representative gel for single cell clones derived from DF-1 cells transfected with the RET-targeting sgRNA-Cas9 and the ssODN for the MEN2B and MEN2A/HSCR HDR modifications respectively. The table shows the ratio of the monoallelic and biallelic HDR-based mutations detected with single cell clones and the overall efficiency in percentage: N = 19 for MEN2B and N = 12 for MEN2A/HSCR.

We validated the activity of the CRISPR/Cas9 system by designing sgRNAs (Supplementary Table 2) targeting the translational initiation region (start codon) of *DROSHA, DICER, MBD3, KIAA1279, CDKN1B* and *EZH2.* We generated NHEJ-induced mutation by co-transfecting the sgRNA CRISPR/Cas9 construct with or without a puromycin resistance-expressing construct into the chicken fibroblastic DF-1 cell line using Lipofectamine 3000 (Fig. 1a). Genomic DNA was isolated after 72–96 hrs and the frequency of induced mutation in the targeted locus was analysed using the T7E1 assay and DNA sequencing (Supplementary Table 3). In puromycin-resistant cells, cleavage bands ranging between 20–68% were visible in all target genes as calculated by Image J software. The mutation efficiency induced was, for example, 50–51% in *KIAA1279*, 26–49% in *CDKN1B*, 68% in *MBD3* (Fig. 1a), 58% in *DICER* and 38% in *EZH2* genes (Supplementary Fig. 1B). We further characterised cleavage by sequencing and this showed different indels detected at all the target sites with various mutation sizes (Supplementary Fig. 1D). We also targeted *KIAA1279*- and *CDKN1B*-with a different transfection method in a different cell line, using electroporation of the chicken B cell DT40 cell line, with similar results (Fig. 1b).

### The CRISPR/Cas9 system precisely edits genes in chicken cell lines

Next, to test whether specific gene editing through HDR could be generated by the CRISPR/Cas9 system, we chose exons 10 and 16 of the *RET* gene which harbour, respectively, the mutation causing Multiple Endocrine Neoplasia 2A (MEN2A) and Hirschsprung disease (MEN2A/HSCR: C620R in humans and C612R in chickens) and MEN2B (M918T in humans and M910T in chickens) (Supplementary Fig. 1A)^26^,^27^. We designed ssODNs with restriction enzyme site creation and disruption and co-transfected the sgRNA-CRISPR/Cas9 construct with the ssODN and the puromycin construct into the DF-1 cell line, using Lipofectamine 3000. Genomic DNA was isolated from 72–96 hrs post-transfected cells and the frequency of HDR-mediated genetic modification was analysed by digesting the PCR product with EcoRV and BamHI restriction enzymes. The digested bands indicate the frequency of HDR-mediated genetic modification from the ssODN template, which ranged between 34–66% of puromycin-resistant cells (Fig. 1c). Single clonal analysis shows the efficiency of biallelic and monoallelic HDR-mediated genetic modification by the CRISPR/Cas9 system as confirmed by gel and sequencing; 75% monoallelic with no biallelic for MEN2A/HSCR clones and 26% monoallelic and 21% biallelic for MEN2B clones (Fig. 1d).

### The CRISPR/Cas9 system mediates larger genomic deletions in chicken cell lines

**Figure 2:**
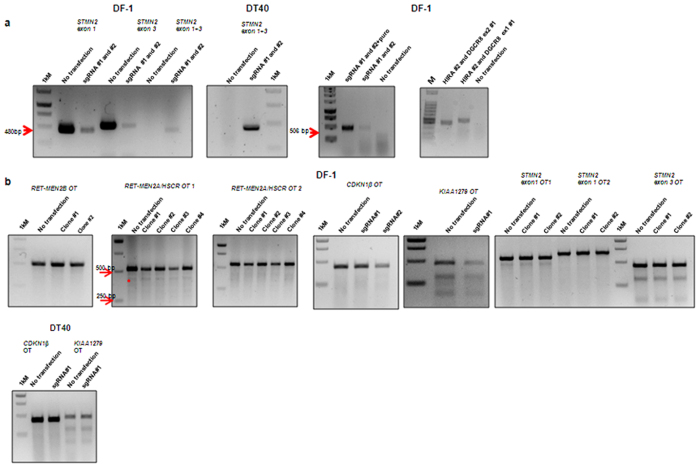
Targeted deletion of large genomic fragments and off-target effect analysis in chicken cells *in vitro*. (**a**) Representative gels showing the large genomic deletions within the *STMN2* locus (>24 kbp) in chicken DF-1 and DT40 lymphoma B cells, and within the *RET* (>8 kbp) and *HIRA-DGCR8* locus (>70 kbp). (**b**) Frequency of off-target effects mediated by RET-MEN2B and MEN2A/HSCR, *CDKN1B, KIAA1279* and *STMN2*-targeting sgRNA-CRISPR/Cas9 system system in chicken DF-1 cells and *CDKN1B* and *KIAA1279* -targeting sgRNA-CRISPR/Cas9 system in DT40 cells by PCR and T7E1 assay. ND-not detected, 1 kM- 1 kbp DNA ladder.

To see whether the CRISPR/Cas9 could also be used for large genomic fragment manipulation, we designed two sgRNAs targeting exon 1 and exon 3 of the *STMN2* gene which spans >24 kilobase pairs (kbps), exon 10 and exon 18 of the *RET* gene which spans >11 kbp, and exon 1 and 2 of *DGCR8* gene and exon 1 of *HIRA* genes which spans >75 kbps of the chicken genome (Supplementary Table 2). We first co-transfected the two sgRNAs in DF-1 cells and analysed the targeted deletion by PCR after genomic DNA extraction. The PCR results (Fig. 2a) shows that the CRISPR/Cas9 system can mediate large genomic deletions (frequency 15%) in chicken cells *in vitro* which is in concordance with published data for other species^18^,^28^,^29^. We then further applied this approach to the chicken DT40 cell line, targeting the *STMN2* locus, and sequencing confirmed the >24 kbp deletion within this locus.

### The CRISPR/Cas9 system produces no detectable off-target effects in chicken cell lines

Off-target mutagenesis remains a draw-back in the use of PENs, and in human cells the CRISPR/Cas9 system has been reported to have relatively high off-target effects compared to other PENs^30^,^31^,^32^. Potential off-target sites with higher scores using the crispr.mit.edu software were selected and analysed by T7E1 assay in both the chicken DF-1 cells and the DT-40 cells of the *KIAA1279* and *CDKN1B*-targeting sgRNA-CRISPR/Cas9 constructs and that of the HDR experiments targeting *RET* in DF-1 cells (Fig. 2b). There were no detectable off-target effects and these results suggest that chicken cells can potentially serve as a model for the use of the CRISPR/Cas9 nuclease, which is known for off-target effects in the human context.

### The CRISPR/Cas9 system can act efficiently without selection in chicken cell lines

Drug selection cannot be used in *in vivo* applications so we investigated whether acceptable gene modification efficiency can be obtained without puromycin selection. sgRNA activity and hence efficiency of mutation-induction can be affected by target locus location, chromatin structure, and nucleotide preferences^33^. Our results show that drug selection for the CRISPR/Cas9 system in chickens is not a necessity but can however improve efficiencies for some sgRNAs with low targeting efficiencies (Supplementary Fig. 1C).

### CRISPR/Cas9 mediates somatic cell modification in chicken embryonic cells *in vivo*

Genetic engineering techniques in chicken have been the genomic modification of PGCs with a germ-line transmission capacity using the lentiviral system^34^,^35^ or Piggybac transposon vector^34^,^36^. Recent work by Park *et al*. showed that TALENs can efficiently generate knockout of targeted genes in chicken cells and in PGCs^4^,^22^. We then tested the efficiency of the CRISPR/Cas9 system in introducing NHEJ mutations into the avian embryo by *in vivo* electroporation. *In vivo* electroporation is a useful tool for the study of spatio-temporal gene functions, since the manipulation of genes can be used to study the roles of such genes in a restricted region during specific developmental stages^4^,^6^.

We injected and electroporated the *DGCR8* exon 2-targeting sgRNA-CRISPR/Cas9 plasmid vectors incorporating mCherry marker *in vivo* into E1.5 embryo cranial neural tube, which transfects the brain and cranial neural crest (Supplementary Fig. 2A). DGCR8 is involved in miRNA processing and the targeted mutation should abrogate the gene function (Supplementary Fig. 2B). We also co-electroporated Tol2-GFP/transposase construct to indicate the trend and variability of transfection where neural crest cells migrate out of the neural tube to surround the brain and eye, and migrate to the branchial arches and facial mesoderm (Supplementary Fig. 2A). Note that for this technique, the distribution and number of transfected cells, as shown by the extent of GFP expressing cells, is variable^37^.

**Figure 3:**
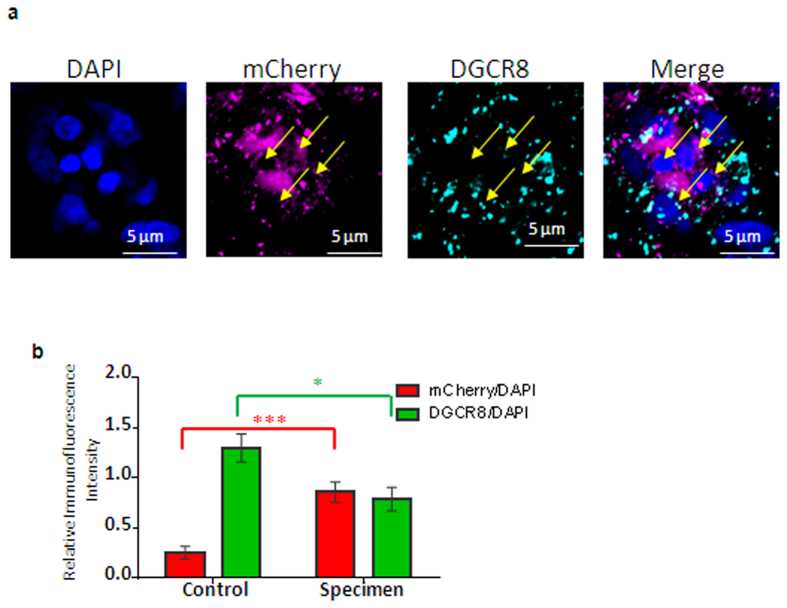
Protein expression 2 days after transfection with *DGCR8* CRISPR/Cas9 construct. (**a**) Immunofluorescence confocal images of single and merged channels of the indicated markers from whole mount staining of *DGCR8* mutant embryos, indicating reduced to no *DGCR8* expression in transfected (mCherry+) cells (shown by yellow arrows). (**b**) Histogram of pixel counts on control embryos and *DGCR8* mutants embryos relative to DAPI. A total of 540 cells and 542 (> = 100 cells/embryo) were counted from 5 control and 6 electroporated embryos respectively. The low fluorescence in the mCherry waveband in controls is tissue autofluorescence. Scale bar: 5 μm. Error bars, mean ± s.e.m. *P < 0.05, ***P < 0.001.

After embryo electroporation with the CRISPR/Cas9 construct, we analysed the hindbrain and midbrain by whole mount immunofluorescence for DGCR8 expression. Since the mCherry plasmid is episomal, expression of mCherry is transient^37^,^38^, embryos were harvested after two days and immunostained with mCherry and DGCR8 antibodies (unelectroporated embryos, N = 8; electroporated embryos N = 24). Electroporated (i.e. mCherry-positive) cells in the midbrain, hindbrain and eye region showed a decrease in DGCR8 expression as determined by decrease in DGCR8 immunofluorescent intensity measured by pixel–count from confocal images (Fig. 3a,b). It is important to mention that DGCR8 expression in postnatal mouse brain has a major nuclear location (Supplementary Fig. 4A), but mainly cytoplasmic location was observed in embryonic chick brain cells (Fig. 3a). Subcellular heterogeneity of location has been reported for DGCR8^39^. In addition DGCR8’s binding partner Drosha also shows cytoplasmic as well as nuclear localisation^40^. The neuronal RNA-binding proteins HUC/D also have different localisation patterns in rodent and chicken cells (compare figures in Hao *et al*.^41^ and Rollo *et al*.^42^). Western blotting of postnatal mouse and embryonic chick brain showed identical DGCR8-immunoreactive protein bands of appropriate molecular weight (Supplementary Fig. 4B), in accord with the high predicted sequence homology between the two species (NCBI database).

In addition, electroporated embryos (N = 34) and control embryos (N = 85) were harvested after four days. The control embryos were used to gauge the background effects of the electroporation technique. The controls were sham-treated non-electroporated embryos (i.e. eggs opened, embryos visualised with India Ink, vitelline membrane nicked and eggs resealed; N = 23), embryos electroporated but without plasmids (i.e. non-transfected; N = 11), embryos electroporated and transfected with the benign Tol2-GFP/transposase plasmids (N = 29), embryos electroporated with the Cas9 construct targeting the unrelated *STMN2* gene (N = 14) and embryos electroporated with the empty Cas9 construct (Cas9 vector with no cloned guide sequence; N = 8).

**Figure 4:**
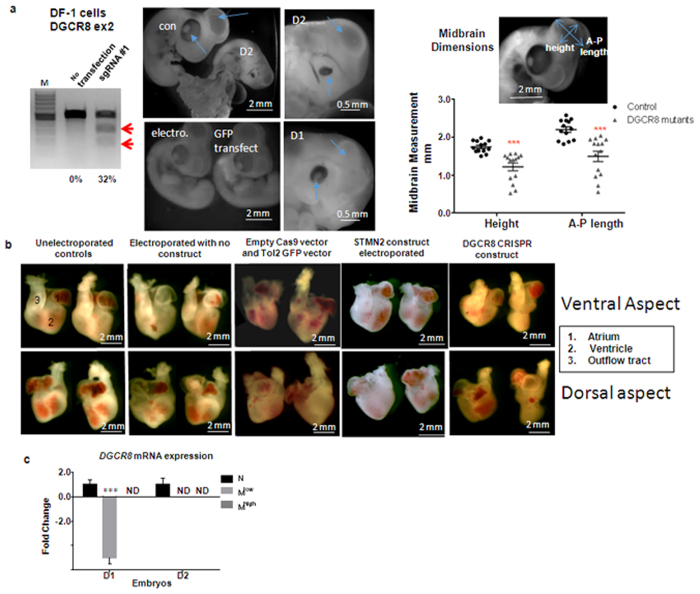
Somatic targeted genetic modification by CRISPR/Cas9 system in chickens 4 days after *in vivo* electroporation. (**a**) Frequency (%) of NHEJ mutation mediated by *DGCR8*-targeting sgRNA-CRISPR/Cas9 system in DF-1 cells by PCR. Red arrows indicate the NHEJ mutation created by the CRISPR/Cas9 system. M- 100 bp DNA ladder. Representative images of sham treated (con), electroporated untransfected embryos (electro.), Tol2 GFP transfected embryos (GFP transfect) with normal head development and *DGCR8* CRISPR/Cas9 transfected embryos (D1 and D2) showing midbrain (open arrow) and eye (closed arrow) abnormalities. Graph shows the difference in the midbrain dimensions of *DGCR8* mutant embryos compared to control embryos- N = 14. (**b**) Representative image of the hearts of unelectroporated embryos, electroporated with no construct embryos, Tol2 GFP and empty Cas9 transfected embryos, *STMN2* transfected embryos (negative control) showing normal heart development, and *DGCR8* transfected embryos showing misshapen and reduced hearts. (**c**) qPCR analysis of cells isolated by FACS from *DGCR8*-targeted embryos demonstrating the reduced mRNA levels of *DGCR8* in mCherry+ brain cells (M) relative to negatively sorted cells (N). Normalisation was done with ACTB and RPL32. ND-not detected. Error bars, mean ± s.e.m. ***P < 0.001.

As expected, after 4 days mCherry fluorescence in wholemount embryos had declined to a level undetectable with the fluorescence stereomicroscope but we could FACs sort cells (mCherry^low^ and mCherry^high^) from the hindbrain. We performed T7E1 and qPCR analysis on these cells and showed a loss of *DGCR8* mRNA levels in mCherry^high^ sorted cells which was confirmed by sequence analysis (Supplementary Fig. 2B). *DGCR8* sgRNA -CRISPR/Cas9 induced nucleotide deletions at the targeted locus with 1–5 bp deletions and insertions. In most clones this formed a stop codon which would result in nonsense-mediated decay of the *DGCR8* mRNA (Supplementary Fig. 2B). The qPCR results coupled with the sequence data suggest a drastic decrease in *DGCR8* gene expression in these brain cells during development, which could delay or disturb the growth of the midbrain as a whole. In addition the expression level of several other genes were analysed with findings consistent with previous findings (Supplementary Fig. 3A)^43^,^44^,^45^,^46^. Of the 34 *DGCR8*-targetted transfected embryos, 8 had a reduced head size and distorted morphology exemplified by major reduction of the midbrain (Fig. 4a). They also showed a reduction in their eyes: it has been reported earlier that miRNAs play an essential role in the differentiation of the retinal pigmented epithelium^47^. Furthermore, we compared the morphology of the hearts of the *DGCR8*-targetted embryos to the control embryos since in the *DGCR8* mutant mouse, decreased *DGCR8* expression results in a spectrum of malformations and reductions in cardiovascular development^48^. We observed deformations in the heart and outflow tract in 14 embryos (41.2%) of the transfected embryos. It is important to note that none of the 85 control embryos had these cranial, retinal and cardiac abnormalities (Fig. 4a,b).

These finding provide further support that the chicken embryo can be genetically modified *in vivo* in a targeted and sophisticated way to study disease models in developing embryos.

## Discussion

The chick embryo is not only an excellent and reproducible system for embryonic developmental studies but also its accessibility and versatility makes it an alternative model in research directly relatable to humans and other animals. We have shown that the CRISPR/Cas9 system can modify multiple genes on both avian macro- and microchromosomes at acceptable efficiency with or without selection, with no detectable off-target effects, a previously mentioned drawback in the use of the CRISPR/Cas9 system^49^.

The function of genes can be spatio-temporally studied using CRISPR/Cas9 system *in vivo* provided that selection is not required. A recent example uses viral delivery to the adult mouse brain^50^. Moreover, refinements to increase the efficiency^51^ and limit the off-target errors^52^,^53^ are progressing rapidly. This means that for developmental studies, the advantages of the chick embryo as an accessible model and the convenience of *in vivo* electroporation at chosen developmental stages and locations can be combined with the power of CRISPR/Cas9 gene editing. We confirm this prediction here, extending the previous trial with *Pax7*^25^.

*In vivo* transfection, including *in vivo* electroporation, affects a modest and variable proportion of cells^37^. This means that, as in many *DGCR8* electroporated embryos, a gross phenotype will not be observed in every instance since gross phenotyppic change depends on high mutational load^54^,^55^. Despite this, functional effects of the genetic modification at the cellular level *in vivo* can be accurately gauged by imaging mutated cells and comparing with non-modified control cells in the same specimen. This requires markers of both the transfected cells and their otherwise similar control cells.

An interesting application of CRISPR/Cas9 editing would be the study of genes involved in nervous system development^56^, organogenesis and structural patterning. An important application would be to modify genes for growth factor response, proliferation or differentiation in neural crest cells. A specific clinically relevant example is the effect of MEN2 mutations which induce a variety of neurocristopathies and developmental cancers^57^. In addition, targeting of primordial germ cells by CRISPR/Cas9 offers the hope of genetically engineering avian models with any desired gene variant.

In conclusion, we have shown that transiently expressing the CRISPR/Cas9 construct can mediate genetic modification of avian embryonic somatic cells, reducing mRNA levels and generating phenotypes in the whole embryo. These results are in congruence with recent work also showing the efficiency of genome editing of postnatal mice using the CRISPR/Cas9 system^58^.

## Methods

### Ethics Statement

All experiments were performed with the official approval from the Murdoch Childrens Research Institute Animal Ethics Committee AEC650 and AEC677 and Institutional Biosafety Committee 226–2015 PC2 NLRD and in strict accordance with its guidelines and those of the Australian Code of Practice for the Care and Use of Animals for Scientific Purposes, 7TH Edition 2004 and the Prevention of Cruelty to Animals Act, Victoria 1986.

### sgRNA-CRISPR/Cas9 system design and construction

Potential target sites were predicted using crispr.mit.edu software in the chicken genome and two to three target sequences with lower predicted score for off-targets were chosen. To construct the sgRNA-CRISPR/Cas9 construct for each target gene, we annealed two complementary 24-bp oligonucleotides (Bioneer Company, South Korea) with the 20-bp target sequence to generate a double-strand DNA with 4-bp overhangs on both ends and cloned into BsaI-digested px330-IRES-mCherry. Oligonucleotides are listed in Supplementary Table 2.

### DF-1 cell culture and transfection

The chicken DF-1 cell^59^ line was maintained and sub-passaged in DMEM (Thermo Scientific), supplemented with 10% fetal bovine serum (FBS; GIBCO) and 1× penicillin/streptomycin (GIBCO), at 37 °C in 5% CO_2_. Cells were seeded at 0.4–0.8 × 10^5^ cells/well in 24-well plates, incubated for 4 hrs, and then transfected with 1.5 μg CRISPR/Cas9 sgRNA targeting the specified gene or region and with or without 0.15 μg of puromycin expression vector using Lipofectamine 3000 (Invitrogen) according to the manufacturer’s protocol with slight modifications. Briefly, 1 μL and 2 μL of Lipofectamine 3000 reagent was added to two different tubes with 25 μL of OPTI-MEM medium (Invitrogen) and briefly vortexed. Then a mixture of 1.5 μg of the CRISPR/Cas9-sgRNA plasmid (px330-IRES-mCherry), 0.15 ug puromycin expression vector and 3 μL of p3000 reagent (Invitrogen) in 50 μL OPTI-MEM was made and then 25 μL of the p3000-DNA complex was then added to each of the Lipofectamine 3000 complex tubes and incubated for 5 minute at room temperature. The complex mixture was then gently pipetted into a well of a 24-well plate with DF-1 cells at about 60–80% confluency. After 24 hrs post-transfection, the cells were treated with puromycin at a final concentration of 2 μg/mL for 2 days and the cells were allowed to recover for a day or two.

For HDR knock-ins, cells were transfected with 1.0 μg CRISPR/Cas9 sgRNA targeting the specified gene or region with 40 pmoles of ssODN and 0.15 μg of puromycin expression vector following the same protocol.

### DT40 cell culture and transfection

Cells of the chicken B cell line DT40^60^ were cultured in chicken medium composed of RPMI-1640 medium (Sigma Aldrich), 10% FBS, 1% chicken serum (Sigma) and penicillin/streptomycin, at 39 °C in 5% CO_2_. A total of 1–2 × 10^7^ cells were pelleted at 1500 rpm for 3 min at room temperature and the pellet was washed with PBS and pelleted again. The pellet was then resuspended in 600 μl PBS and after the addition of 30 μg of sgRNA-CRISPR/Cas9 plasmid and 3 μg of puromycin expression vector, the resuspension was placed in a BioRad 4 mm electroporation cuvette. Electroporation was done using BioRad Gene Pulser II at 250 V and 950 μF. After electroporation, cells were mixed with 10 ml of culture medium without penicillin/streptomycin and cultured for 12–24 hrs. The cells were treated with puromycin at a final concentration of 2 μg/mL for a day and the cells were allowed to recover for 2–3 days.

### T7E1 mutation frequency analysis

Samples of cells and embryos were collected and digested in nuclear lysis buffer (Promega, Madison, WI). Genomic DNA was extracted from DF-1 cells or DT40 cells after transfection of each CRISPR/Cas9-sgRNA. The genomic DNA was extracted from the lysate by phenol-chloroform and recovered by isopropanol precipitation. The genomic region encompassing the CRISPR/Cas9 target site was amplified with a specific primer set for each gene (Supplementary Table 3). The amplicons were re-annealed to form a heteroduplex DNA structure after denaturation and then treated with 2.5 units T7E1 (New England Biolabs, Ipswich, MA) for 20 min at 37 °C and then analyzed by 2% agarose gel electrophoresis. Mutation frequencies were calculated as previously described^61^ based on the band intensities using Image J software and the following equation: mutation frequency (%) = 100 × (1 −(1 −fraction cleaved)1/2), where the fraction cleaved is the total relative density of the cleavage bands divided by the sum of the relative density of the cleavage bands and uncut bands. To confirm target locus mutation, PCR amplicons were cloned into a pGEM-T Easy vector (Promega, Madison, WI) and sequenced. Primers for the PCR analysis are listed in supplementary figures.

### Restriction Enzyme digestion (RFLP)

Six μl of the PCR product was digested with 0.5 μl of the required restriction enzyme in a 20 μl reaction and incubated for 4–8 hrs at 37 °C. For MEN2B HDR templates, the PCR products were digested in BamHI restriction enzyme (New England Biolabs, Ipswich, MA) and in EcoRV restriction enzyme (New England Biolabs, Ipswich, MA) for MEN2A/HSCR HDR experiments. Digested products were then analysed by 1.5% agarose gel electrophoresis. Positive clones were cloned into the pGEM-T Easy vector (Promega, Madison, WI) and cloned products were sequenced.

### Sequencing analysis

PCR products were cloned into the pGEM-T Easy vector (Promega, Madison, WI) and cloned products were sequenced using the T7 promoter primer (5′-TAATACGACTCACTATAGGG-3′).

### Off-target prediction and analysis

Potential chicken *KIAA1279, CDKN1B, STMN2* and *RET* gene off-targets were predicted using crispr.mit.edu software in the chicken genome. Off-target site with scores more than 1 or with 2 or more mismatches were chosen and amplified by PCR using the extracted genomic DNA as templates. The PCR products were first subjected to T7E1 cleavage assay^14^. Oligonucleotides are listed in Supplementary Table 4.

### Single cell clonal analysis

Cells were trypsinized and plated in 96 well plates at average 0.3 cell/well and incubated at 37 °C for two weeks. Each well was then microscopically evaluated, and single cell-derived clones were selected and expanded into 24 well plates. Genomic DNA from each clone was extracted and T7E1 assay was conducted following the above protocol. To confirm HDR of the ssODN, PCR amplicons were digested with 5 units of the restriction enzyme BamH1 (New England Biolabs) for more than 2 hrs at 37 °C and then analysed on 2% agarose gel by electrophoresis. PCR amplicons of BamH1 or T7E1 digested clones were cloned into a pGEM-T Easy vector (Promega, Madison, WI) and sequenced.

### *In vivo* electroporation

Eggs from a cross breed *White Leghorn x Black Australorp* were commercially purchased (Research Hatchery, Victoria). *In vivo* electroporation were performed as previously described^37^,^62^,^63^. The CRISPR/Cas9 *DGCR8* exon 2 targeting construct was co-electroporated with pT2K-CAAGGS-EGFP (termed Tol2-GFP) and pCAGGS-T2TP (transposase)^38^ at 6:6:1.5 μg/μL ratio respectively. The plasmid mixture was prepared and coloured with 2% Fast Green and then microinjected forward from the 3–4 somite level of the neural tube into the hind and midbrain of E1.5 chicken embryos. Electric pulses of 10.5 V, 50 ms duration were delivered 3 times bilaterally with 175-ms intervals. Chicken embryos were harvested 2 and 4 days post-electroporation and processed for immunostaining and cell sorting.

### Fluorescence Activated Cell Sorting

Cell suspensions from harvested embryos were made using 0.5% w/v Dispase II (Roche, Switzerland) and 0.1% *w/v* CLSAFA Collagenase (Worthington, USA) at 37 °C in Hams F12 solution for 5–10 minutes. The digested cells were pelleted and resuspended in PBS containing 2% FBS and strained (40 μm mesh; BD Falcon; Becton, Dickinson and Co., Franklin Lakes, NJ) and FACS sorted using the BD Influx Cell Sorter, with separation based on GFP and mCherry fluorophores.

### RNA extraction and SYBR Green qPCR

Total RNA was extracted from sorted cells using the Trizol reagent (Invitrogen) and lysate were purified by the acid-phenol chloroform and recovered by isopropanol/ethanol precipitation method. Extracted RNA was digested with DNaseI (Promega) following the manufacturer’s instructions to remove any residual DNA.

qPCR was performed to confirm the expression of *DROSHA, Neurogenin 2 (Ngn2), Pax6, YPEL1*, and *DGCR8* genes in transfected modified embryonic cells from electroporated embryos. Briefly, 20 ng total RNA was converted into cDNA in the presence of SuperScript IV RT (Invitrogen) and random hexamers (Promega). Reactions were performed using cDNA converted from 10 ng of RNA, 250 nM of each primer and 2X SYBR Green qPCR Master Mix (Promega) in a total volume of 20 μl. Primers for qPCR analysis are listed in Supplementary Table 5. *ACTB* and *RPL32* were used for data normalization. mCherry-/GFP- sorted cells from each embryo were used as a calibrator and relative fold changes were calculated using the 2−[Δ][Δ]Cq method.

### Whole mount staining and immunohistochemistry

Embryos were harvested 2 days post electroporation (E3.5) and sagittally dissected, fixed in 4% paraformaldehyde in PBS at 4 °C overnight then washed in PBS three times. Embryos were blocked and permeabilised with 3% horse serum and 0.2% Triton-X100 in PBS/azide for 1 hr. Control cryostat 18 μm sections of chick embryo and post-natal mouse brain were also used. Rabbit anti-DGCR8 antibody (Abcam-ab82876) and mouse anti-mCherry (DSHB, Iowa City) at 1:200 and 1:100 respectively were applied in 1% horse serum and 0.1% Triton-X in PBS azide and incubated on a rocker at 4 °C overnight. The human *DGCR8* immunogen (N-terminal amino acids 180–229) was 89% identical to the predicted chick amino acid sequence (NCBI database), with all changes conservative. Washing with PBS was done for 3 hrs with changes every 30 minutes on a rocker at 4 °C. Secondary antibodies were donkey anti-rabbit:Alexa Fluor 488 for mouse sections (Life Technologies-1:1000) and donkey anti-rabbit:Cy5 plus donkey anti-mouse:Alexa Fluor 568 for whole mounts (Jacksons Immunoresearch-1:500 and 1:1000 respectively) and 500 ng/mL (1 in 100 of 50 ug/mL stock) DAPI was applied and incubated for 3 hrs on the rocker at 4 °C. Embryos were washed with PBS three times and mounted using DABCO/glycerol mounting medium. Confocal microscopy was performed using the Zeiss LSM 780 confocal microscope.

For cryostat sections, fixed embryo heads (E5.5) were placed in 30% sucrose in PBS overnight, embedded in Tissue Tek OCT Compound Medium in Tissue Tek cryomoulds (both from ProSciTech, Thuringowa, Australia) and frozen in dry ice-cooled isopentane. Eighteen μm sections were cut transversely using a Leica CM 1900 cryostat microtome and collected on Superfrost microscope slides (Biolab Scientific, Auckland, NZ) coated with poly-l-lysine. Mouse post-natal brains were also fixed, sectioned, mounted and stained as above.

### Western Blotting

Brains from chick embryos (E6) were homogenised in 2 mL of sample buffer for SDS-PAGE. Homogenates were sonicated for 40 sec and centrifuged at 13,000 rpm for 5 min. The resulting supernatants were divided into 100 mL aliquots and stored at −80 °C. Protein concentration of 3.3% homogenates (w/v) in PBS was determined by the Pierce BCA Protein Assay Kit (ThermoFisher Scientific) using bovine serum albumin as a standard. Forty micrograms of each sample were analyzed by Western blotting. The apparent molecular mass of *DGCR8* was estimated by a prestained protein marker (Life Technologies). Control tissue was obtained from post-natal mouse brain.

### Relative Pixel Quantification

Standard confocal images were selected and analysed using the Zeiss Image Analyser. Regions of interest were selected with the Free-Hand tool and choosing the DAPI channel, the cells in a field were counted and relative pixel quantification was calculated following a previous publication^64^. Relative fluorescence intensity was calculated by normalising the mCherry and *DGCR8* expression to DAPI intensity.

### Statistical analyses

Data were analyzed by the unpaired t test with Welch’s correction. Values were expressed as mean ± SEM. Changes were deemed significant if the p value was >0.05. Statistical significance is indicated as follows: *p > 0.05, **p > 0.01, and ***p > 0.001. Graphs were drawn using Microsoft Excel and GraphPad Prism.

## Additional Information

**How to cite this article**: Abu-Bonsrah, K. D. *et al*. CRISPR/Cas9 Targets Chicken Embryonic Somatic Cells *In Vitro* and *In Vivo* and generates Phenotypic Abnormalities. *Sci. Rep.* **6**, 34524; doi: 10.1038/srep34524 (2016).

## Supplementary Material

Supplementary Information
