# Supplementary material for: CRISPR/Cas9 Targets Chicken Embryonic Somatic Cells In Vitro and In Vivo and generates Phenotypic Abnormalities
Source: Sci Rep. 2016 Oct 3;6:34524. doi: 10.1038/srep34524 (PMC5046125; doi:10.1038/srep34524)
Supplement: Supplementary Information [file srep34524-s1.pdf]

CRISPR/Cas9 Targets Chicken Embryonic Somatic Cells *In Vitro* and *In Vivo* and generates Phenotypic Abnormalities.

Kwaku Dad Abu-Bonsrah[1,2], Dongcheng Zhang[1,2] and Donald F. Newgreen[2]

[1]Department of Paediatrics, University of Melbourne, Parkville 3052, Australia

[2]Murdoch Childrens Research Institute, Royal Children's Hospital, Parkville, 3052, Australia

Supplementary Table 1: Genes mutated or detected in this study

Gene	Chicken c'some	Type, Function	Developmental expression/role	Human or rodent disease relationship	Present study
CDKN1B	1	Cyclin-dependent kinase inhibitor.<br>Mediates G1 arrest.	Exp. ubiquitous.<br>Leydig cell dev.	MEN4	NHEJ deletion DF-1 ±puromycin *in vitro.*<br>Off-target screen DF-1, DT40 *in vitro.*
CNN1 Calponin1	30	Neg reg by DROSHA.	Cytoskeletal -related	Severe atrioventricular septal defects	Exp. level after *DGCR8* NHEJ deletion *in ovo.*
DGCR8	15micro	Complex & co-reg with DROSHA.<br>miRNA processing.	Exp. wide esp. in neuroepithelium, palate, thymus, limbs.	DiGeorge critical region<br>Het. reduced cell prolif^{n} and neurogenesis	Large transgenic deletion DF-1, DT40 *in vitro.*<br>NHEJ deletion *in ovo.*
DICER	5	Ribonuclease III.<br>miRNA processing.	Exp. ubiquitous esp. facial, pharyngeal arches and limb bud..	Up-reg. in melanoma<br>Down-reg in breast carcinoma	NHEJ deletion DF-1 *in vitro.*
DROSHA	2	Ribonuclease III. miRNA processing. Complex & co-reg with DGCR8	Exp. ubiquitous esp. viscera.	Premature death<br>Cachexia	NHEJ deletion DF-1 *in vitro.*<br>Exp. level after *DGCR8* NHEJ deletion *in ovo.*
EZH2	2	Polycomb family.<br>Regulation DNA methylation.<br>Histone modification.	Exp. neuroepithelium, pharyngeal arches, myotome, later esp. testis.<br>Lung, CNS and B cell dev.	Weaver syndrome	NHEJ deletion DF-1 ±puromycin *in vitro.*
HIRA	15micro	Histone cell cycle regulator.	Exp. esp. pharyngeal arches.	DiGeorge critical region	NHEJ deletion DF-1 ±puromycin *in vitro.*<br>Large transgenic deletion DF-1 *in vitro.*
KIAA1279 (KIF1BP)	6	Regulation DNA methylation.<br>Histone modification.	Exp. ubiquitous.	Goldberg-Shprintzen Hirschsprung	NHEJ deletion DF-1 ±puromycin *in vitro.*<br>Off-target screen DF-1, DT40 *in vitro.*<br>Exp. level after *DGCR8* NHEJ deletion *in ovo.*
NGN2	4	Regulated by DROSHA/DGCR8.	Exp. in neural tube, pharyngeal arches	PNET	Exp. level after *DGCR8* NHEJ deletion *in ovo.*
PAX6	5	Paired box homeotic TF.<br>Regulated by DROSHA /DGCR8.	Exp. in neural tube, pharyngeal arches, eye.	Aniridia	Exp. level after *DGCR8* NHEJ deletion *in ovo.*
MBD3	28micro	NuRD complex subunit<br>Chromatin alteration, epigenetic markers.	Exp. wide esp. in brain, pharyngeal arches, intestine	Siver-Russell syndrome	NHEJ deletion DF-1 ±puromycin *in vitro.*<br>Exp. level after *DGCR8* NHEJ deletion *in ovo.*

RET	6	GF receptor tyrosine kinase	Exp. in PNS, CNS, kidney.<br>Survival, proliferat^{n}, different^{n}.	MEN2A, MEN2B, Hirschsprung	HDR gene editing C612R, M918T DF-1 *in vitro.*<br>Large intragenic deletion DF-1 *in vitro.*
STMN2	2	Target of MAPK8<br>Regulation of microtubule stability	Exp. CNS and PNS.<br>Neuronal growth.	Alzheimer	Large intragenic deletion DF-1, DT40 *in vitro.*<br>NHEJ deletion *in ovo.*<br>Exp. level after *DGCR8* NHEJ deletion *in ovo.*
TYRP1	Z	Tyrosinase-related protein-1, melanosomal tyrosinase	Exp. epidermal NCC, retina.<br>Melanin biosynthesis.	Rufous oculocutaneous albinism	NHEJ deletion DF-1 ±puromycin *in vitro.*
YPEL1	15micro	Centrosomal/nucleolar<br>Regulated by DROSHA /DGCR8	Exp. in pharyngeal arches.	DiGeorge critical region	Exp. level after *DGCR8* NHEJ deletion *in ovo.*

Gene data from: http://www.ncbi.nlm.nih.gov/gene/

Expression data from:

http://www.proteinatlas.org/search/

http://www.emouseatlas.org/emagewebapp/pages/emage_gene_browse.jsf

Disease data from: http://www.genecards.org/cgi-bin/carddisp.pl?gene=

miRNA target data: http://www.targetscan.org

http://www.microrna.org

Supplementary Table 2: Oligonucleotides for cloning sgRNA into CRISPR/Cas9 vector

Gene or locus		Direction	Sequence 5' to 3'
RET exon 16	sgRNA #1	FP1	CCGGAGGGTCGGATACCTGTTAAA
		RP1	AAACTTTAACAGGTATCCGACCCT
	sgRNA #2	FP2	CCGGTCTATGGCCATCCATTTAAC
		RP2	AAACGTTAAATGGATGGCCATAGA
RET exon 10	sgRNA #1	FP1	CCGGTCACAATAACAGTTCTGTCT
		RP1	AAACAGACAGAACTGTTATTGTGA
KIAA1279	sgRNA #1	FP1	CCGGGCGGAACTTCTCGCACGCCG
		RP1	AAACCGGCGTGCGAGAAGTTCCGC
	sgRNA #2	FP2	CCGGCGGCGGCGACAAGATGGCGG
		RP2	AAACCCGCCATCTTGTCGCCGCCG
MBD3	sgRNA #1	FP1	CCGGGCACCTTTGACTTCCGCACG
		RP1	AAACCGTGCGGAAGTCAAAGGTGC
	sgRNA #2	FP2	CCGGGTCAAAGGTGCTCAGGTCCA
		RP2	AAACTGGACCTGAGCACCTTTGAC
EZH2	sgRNA #1	FP1	CCGGACACGTTTTCGCCAACAAAT
		RP1	AAACATTTGTTGGCGAAAACGTGT
	sgRNA #2	FP2	CCGGCTGAGAAAGGACCAATTTGT

		RP2	AAACACAAATTGGTCCTTTCTCAG
CDKN1β	sgRNA #1	FP1	CCGGCCCTACCCTGGAGCGCATGG
		RP1	AAACCCATGCGCTCCAGGGTAGGG
	sgRNA #2	FP2	CCGGCCGCGCCTCCATGCGCTCCA
		RP2	AAACTGGAGCGCATGGAGGCGCGG
Stathmin-like 2 exon 1	sgRNA #1	FP1	CCGGATTGCTGTTTTAGCCATGGT
		RP1	AAACACCATGGCTAAAACAGCAAT
	sgRNA #2	FP2	CCGG AGCGCCTGCACATCCCACCA
		RP2	AAACTGGTGGGATGTGCAGGCGCT
Stathmin-like 2 exon 3	sgRNA #1	FP1	CCGGTGAGGAGATCCAGAAAAAGC
		RP1	AAACGCTTTTTCTGGATCTCCTCA
	sgRNA #2	FP2	CCGGCGGCAGCCTCCAGCTTTTTC
		RP2	AAACGAAAAAGCTGGAGGCTGCCG
DGCR8 exon 2	sgRNA #1	FP1	CCGGGTCTTCGTGCACCCAAAACA
		RP1	AAACTGTTTTGGGTGCACGAAGAC
	sgRNA #2	FP2	CCGGCACTTGTTTCTCCATCAGAC
		RP2	AAACGTCTGATGGAGAAACAAGTG
HIRA	sgRNA #1	FP1	CCGGTATGGCTCCTGTCCTGAAAG
		RP1	AAACCTTTCAGGACAGGAGCCATA
TYRP1	sgRNA #1	FP1	CCGGACCATTGAGTCTCTGAGAAG
		RP1	AAACCTTCTCAGAGACTCAATGGT

Supplementary Table 3: Primers for On-Target analysis.

Gene or locus	Direction	Sequence 5' to 3'
RET exon 16	FP	AACAGATCATCTGTCAGCTTGTGT
	RP	TCCTCAGATTTAATTAAATGGCA
RET exon 10	FP	CTACTCCAGTGCTCAGAAATCCAT
	RP	GTCTCCATGCACAAGTCAATATGC
KIAA1279	FP	GCCAGGGCCCTCGAGTAACCGCG
	RP	GCGCAGCCCCCGCCCACCTGGGC
MBD3	FP	TGCACTTATTCTGCTAATTATGCTG
	RP	TGGGACAGGCTTTGTTCTTGAGA
EZH2	FP	ATGGCCTGTTAATTTGTAACTAAAAT
	RP	TTGATTCAATCACATACAAATGTA
CDKN1B	FP	TCGGCTGCTCCCCTCCGGAGCCG
	RP	GCCTGAAGTAGAAGTCGGGCGAG
Stathmin-like 2 exon 1	FP	GTGGATCAATATTTAATGCCCGGAG
	RP	TGATCTCAGATCGTTAAAAGAGCC
Stathmin-like 2 exon 3	FP	TAGTCTGTATTTCTGATCGTACACT
	RP	CAAGTTGCTGAAAGAAAATAAACT
DGCR8 exon 2	FP	GGGATCATTGGACATTTCTAGTTC

	RP	ACAACAGATGAAGACAAAAAGCAC
HIRA	FP	TGTAACTGTATCAGAATGGCCTCT
	RP	CCAGAGAAAGTTCACTGATTTCCA
TYRP1	FP	CTGGACTGGAGGTTTGTATTTCTT
	RP	CTAGGTGCATTAAAATCAACATGC

Supplementary Table 4. Primers for Off-target Analysis and their Off-target sequence. Mismatch nucleotides are in red

Gene or locus	Off-Target sequence	Direction	Sequence 5' to 3'
RET exon 16 OT	AGGGTCAGCCACCTGTTAAAGGG	T7 FP	AGCACTCGAGTCAGGAGACTTAAT
		T7 RP	ATTGGAGGACTTCTTGTCTAGGTG
RET exon 10 OT1	TCAAAATGACAGTTCTGTCTCAG	T7 FP	GAGAAACAGGAAAATAGGAACAGG
		T7 RP	CAGCTACTGTGACACTTGATTCAG
RET exon 10 OT2	CCATCATAACAGTTCTGTCTCAG	T7 FP	GACCTCAGCATAATCACATTGTTC
		T7 RP	TATCCTATGCTTTACACTCGTCCA
KIAA1279 OT	GTGGAACTTCTCGCACGCAGCGG	T7 FP	AAATAGGGGAGAAAAATGAGGAAG
		T7 RP	AATCCCGCTGGTAAAATATGTATG
CDKN1β OT	CAGCAACCTGGAGCGCATGGGAG	T7 FP	CAAGGTCACAAAGTCTCATTGTTC
		T7 RP	CGTTGTCTTCATCCTTAGGTTTTT
Stathmin-like 2 exon 1 OT1	AGCTCCTGCACATCCCACCTGGG	T7 FP	TTTAAAAGGTAGTCCTGCTGCTCT
		T7 RP	GAGATTTTGCAGTTTCACAGAAGA
Stathmin-like 2 exon 1 OT2	ATCGCCACCACATCCCACCAAGG	T7 FP	TCCACGAAAGTATGCTGAGATAAA
		T7 RP	CTGGGAAAAGGATTACATTCAGAG
Stathmin-like 2 exon 3 OT	TTATAAGATTCAGAAAAAGCTGG	T7 FP	CACCCACAGTCAACTTATTTTTGT
		T7 RP	AAATGAGAGTTCCACAATGCAGTA

Supplementary Table 5: Primers used in qPCR

Gene or locus	Direction	Sequence 5' to 3'	Product size
DROSHA	FP	CATGGATCAAGTGGGGGACT	100 bp
	RP	GGCCTTTGCCTGTCGTTTTC	
NeuroG2	FP	CACAACTACATCTGGGCGCT	175 bp
	RP	GCGATAAAGTGCAGGCGTAG	
DGCR8	FP	GCATGAATATATGCAACGAGTCC	75 bp
	RP	GGCTCACTTGGGTTCTCACA	
ACTB	FP	GTATGTGCAAGGCCGGTTTC	92 bp
	RP	AACCATCACACCCTGATGTCT	
RPL32	FP	GTTACGACCCATCAGCCCTTG	93 bp
	RP	CATGATGCCGAGAAGGAGATGG	
PAX6	FP	GCGCAGTATAAACGAGAGTGC	187 bp
	RP	TCAGCATCCTTAGCTTGTCGT	
YPEL1	FP	GAGCCCACCTAGCCAATCAC	132 bp
	RP	ACAGCCCACATTTACCACAG	
STMN2	FP	CGAGGAGAGGAGAAAGTCCCA	150 bp
	RP	TGTTCCATTTTCAGTATCAGCTTT	
KIAA1279	FP	CTACATCCAGGCCCAGAACA	141 bp
	RP	CTGGGATCCAGGGGAGGATT	

MBD3	FP	TCCAACCAAGCCAAAGGCAA	125 bp
	RP	GGATCACTCTTCACCTTATTGCTG	
CNN1	FP	ATAAGCTGGAGAACATTGGGAAC	97 bp
	RP	GTGTTCTCGAAGAGGTCGTTG	

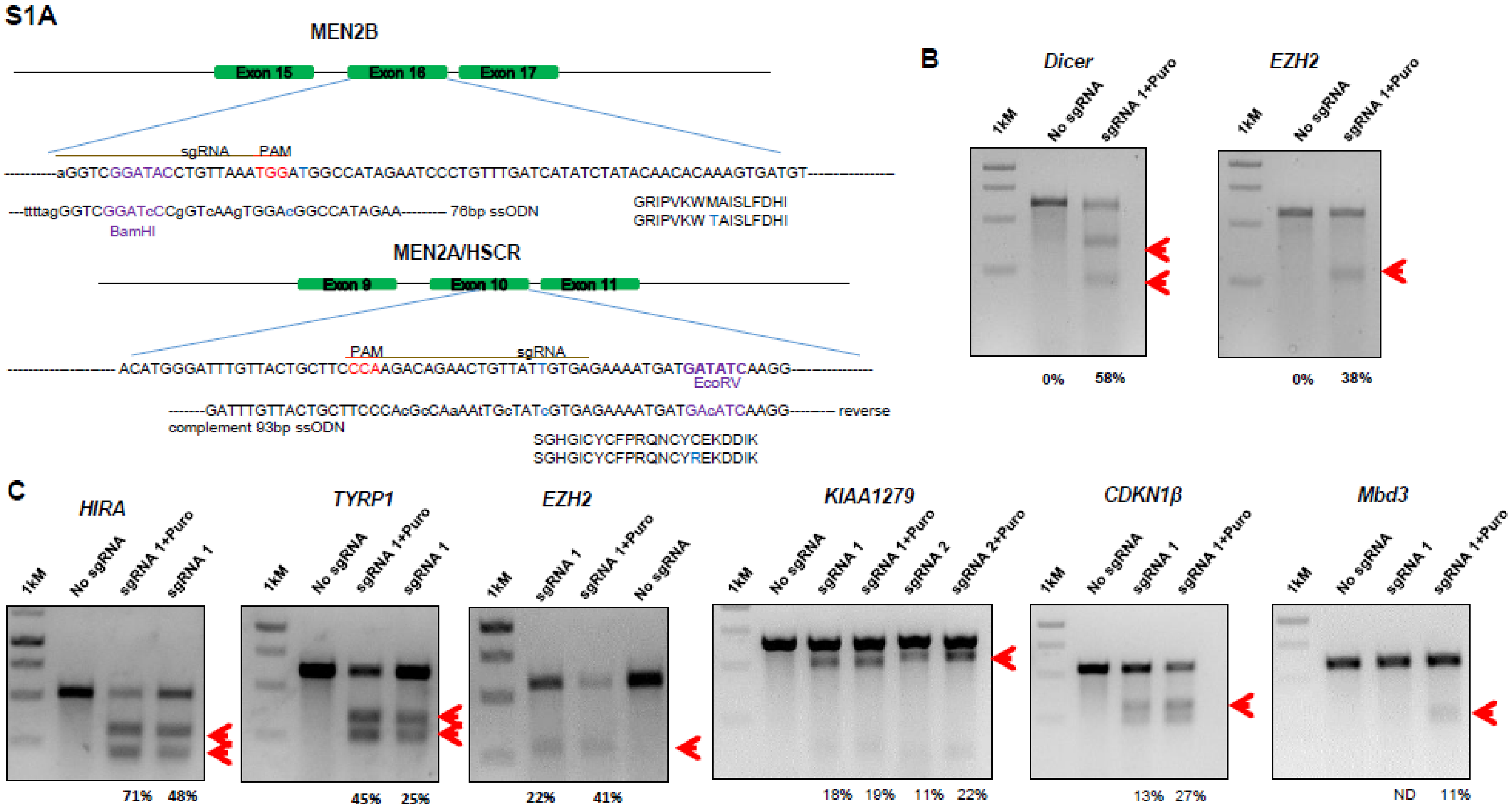

Supplementary Figure 1A. Diagram of sgRNAs targeting sites on exon 10 (MEN2A/HSCR) and exon 16 (MEN2B) of the *RET* gene and the corresponding ssODN. Exons are shown in green boxes with location of sgRNA and sequences of PAM in red. Created (BamHI) and disrupted (EcoRV) restriction enzyme site are in purple. Desired base pair correction and its corresponding amino acid is represented in blue. (**B**) Frequency (%) of NHEJ mutations mediated by *Dicer* and *EZH2*-targeting sgRNA-Cas9 system in DF-1 cells with puromycin selection, by PCR and T7E1 assay. (**C**) Frequency (%) of NHEJ mutations mediated by *HIRA*, *TYRP1*, *EZH2*, *KIAA1279*, *CDKN1β*, and *Mbd3*-targeting sgRNA-Cas9 system in DF-1 cells with and without puromycin selection by PCR and T7E1 assay. Arrow indicates the NHEJ mutation created by the CRISPR/Cas9 system. 1kM-1 kbp DNA ladder. ND=not detected.

S1D

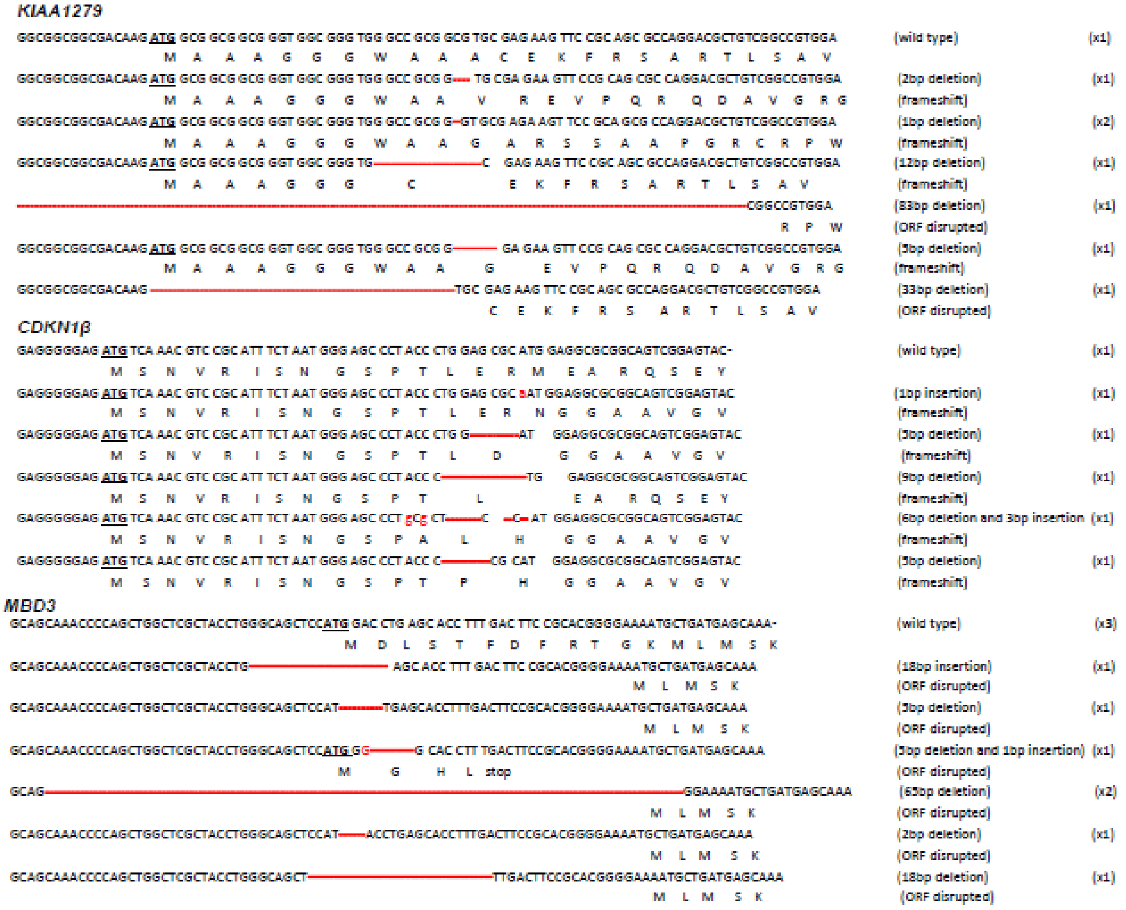

Supplementary Figure 1D. NHEJ mutations in *KIAA1279*, *CDKN1β* and *Mbd3* mediated by sgRNA-Cas9 in DF-1 cells. Red dashes and letters in red in the DNA sequences denote deleted nucleotides and inserted nucleotides respectively, and bold and underlined ATG indicates the translational initiation codon.

S1E

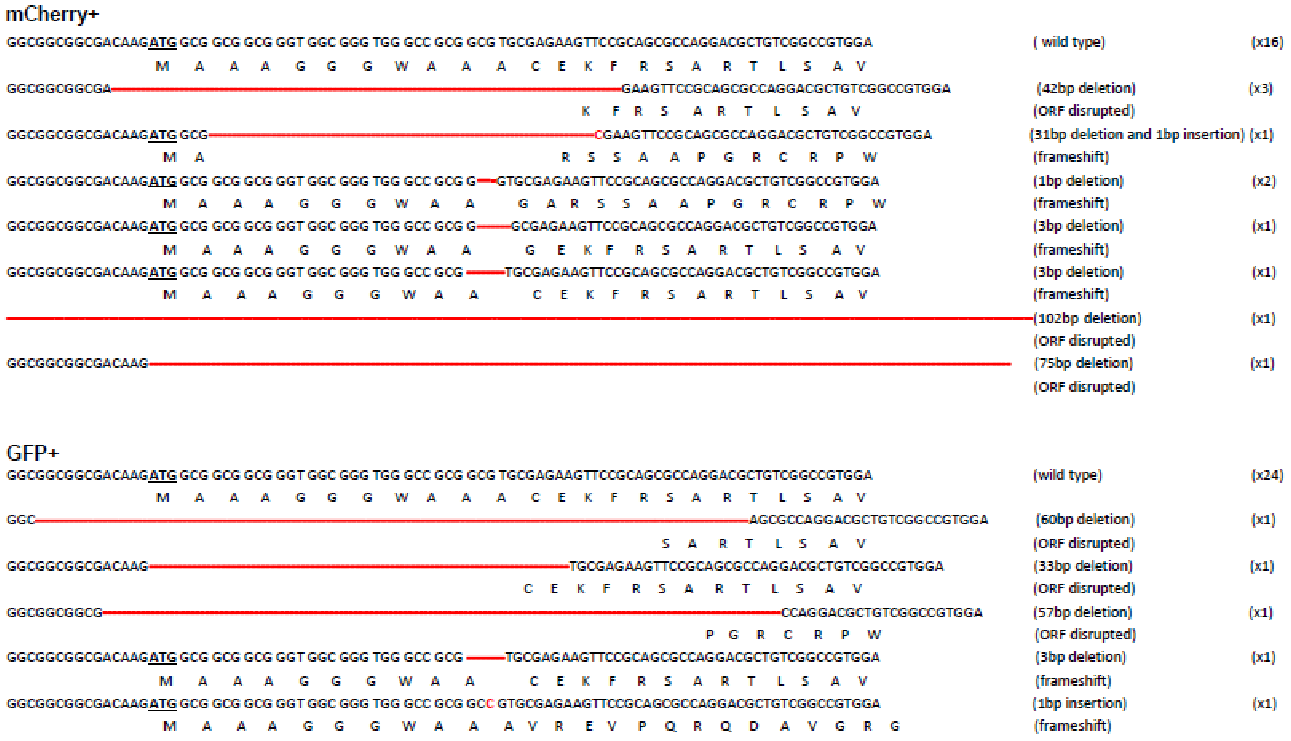

Supplementary Figure 1E : Sequence analysis of NHEJ mutation mediated by *KIAA1279*-targeting sgRNA-Cas9 system in chicken cells *in ovo*. Red dashes and letters in red in the DNA sequences denote deleted nucleotides and inserted nucleotides respectively, and bold and underlined ATG indicates the translational initiation codon in the gene.

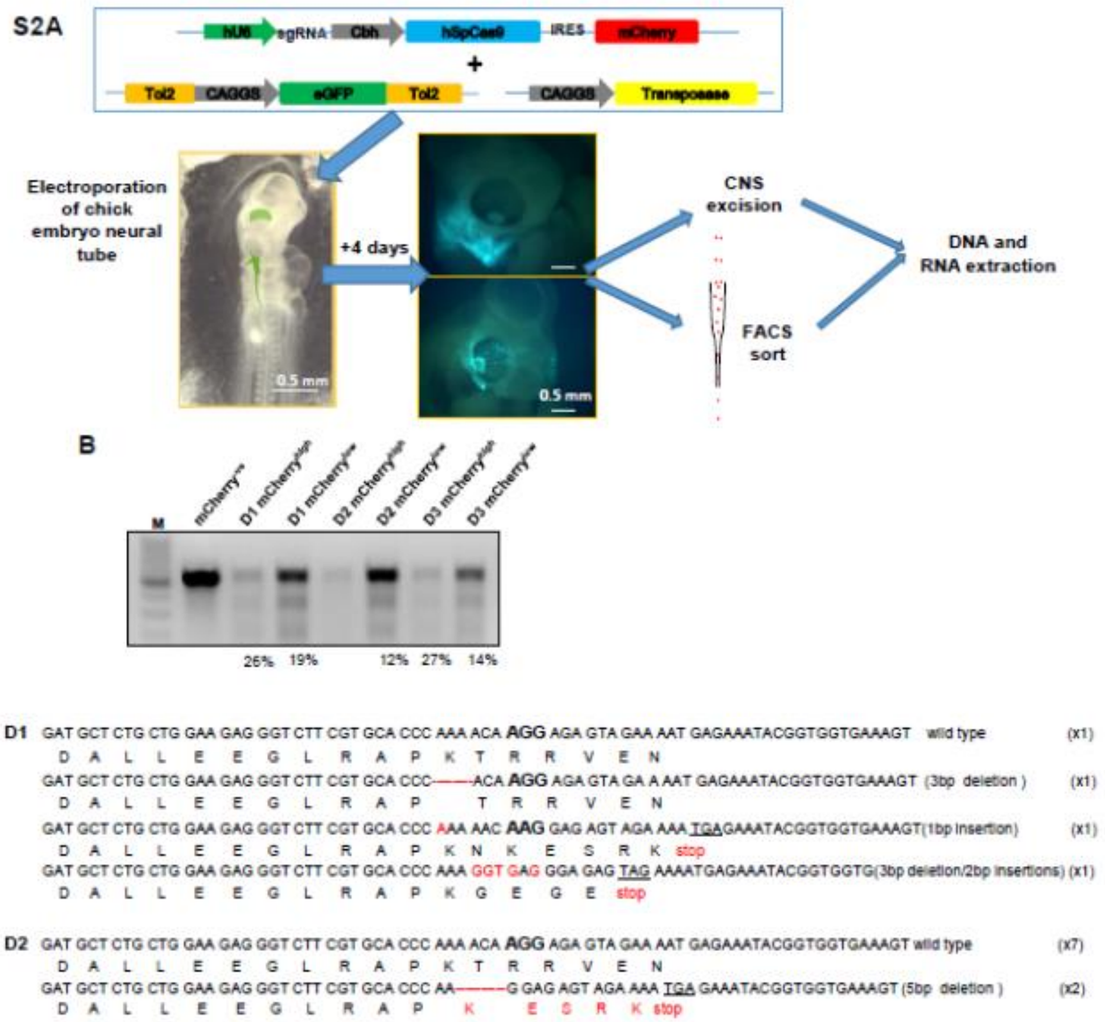

Supplementary Figure 2 (A) A schematic representation of the *in ovo* electroporation technique and FACs. The mCherry CRISPR/Cas9 and the Tol2-GFP/transposase plasmids were co-injected or injected separately into the neural tube lumen (shaded green) of E1.5 chick embryos and electroporated. Embryos were harvested at stage E5.5, dissociated by collagenase/dispase and sorted by FACs or, alternatively, surrounding tissues were removed leaving the CNS for analysis. Representative images showing the extent of the electroporation and distribution of GFP-labelled cells was variable. **(B)** Frequency of NHEJ mutation mediated by exon 2 of DGCR8-targeting sgRNA-CRISPR/Cas9 system in chick embryo by PCR and T7E1 assay and sequence analysis of cloned DGCR8-1 (D8-1) and D8-2 sorted cells. Red dashes and letters in red in the DNA sequences denote deleted nucleotides and inserted nucleotides respectively, and bold indicates the PAM sequence with underlined nucleotide indicating the stop codon. mCherry^{low} – mCherry low, mCherry^{high} –mCherry high, mCherry^{-ve} -mCherry negative, 1M- 100 bp DNA ladder, ND=not detected.

S3A

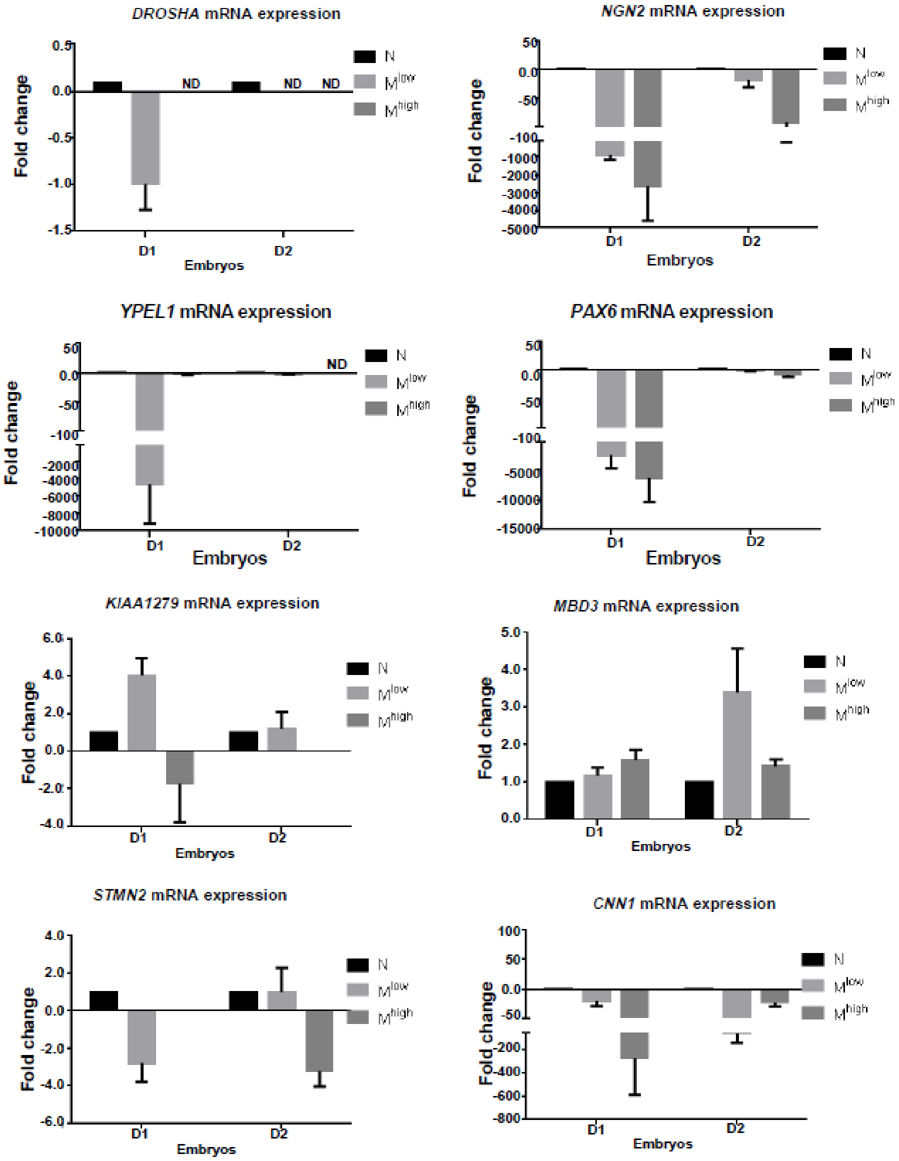

Supplementary Figure 3 (A) qPCR analysis of *Dgcr8*-targeted embryos demonstrating the reduced mRNA levels of neural (*Neurogenin1, Pax6*), cardiac outflow tract (*Ypel1*), smooth muscle cell *(CNN1- positively regulated by DROSHA)* genes in *Dgcr8*-targeted cells (ie. mCherry positive), relative to negatively sorted cells. Normalisation was done with *ACTB* and *RPL32*. N=mCherry negative cells, M^{low}=mCherry low expressing cells, M^{high}=mCherry high expressing cells, **(B)** As a negative control to *Dgcr8* mutant experiments, we electroporated embryos with the two sgRNA-Cas9 construct targeting the *Stmn2* gene. The hindbrains were harvested and extracted genomic DNA and total RNA were analysed by PCR and RT-PCR respectively. Gel shows the frequency (%) of NHEJ mutation mediated by *Stmn2*-targeting sgRNA-Cas9 system in the hindbrain of chicken embryos by PCR and an RT-PCR analysis of *Stmn2* mRNA levels of controls and samples. The chicken β-actin housekeeping gene was used as a reference point. ND- non detectable.

3B

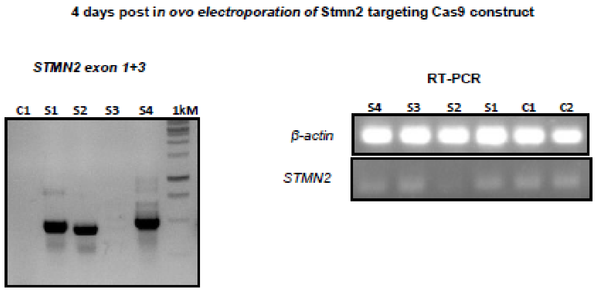

S4A

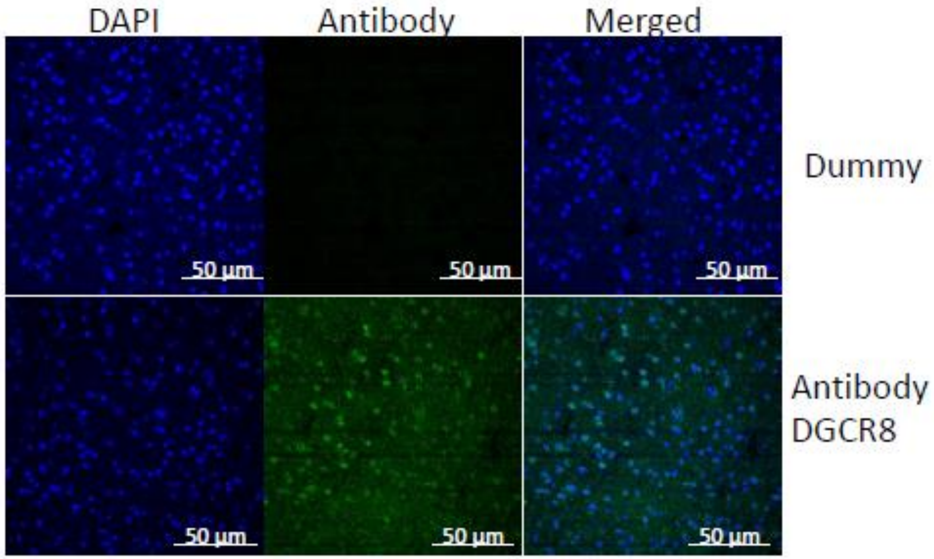

S4B

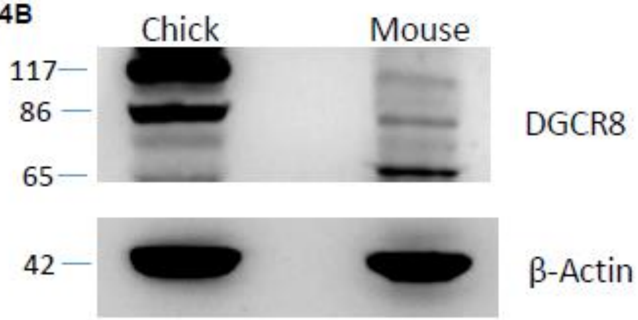

Supplementary Figure 4 (A) Immunofluorescence images of single and merged channels of the indicated markers from frozen sections of postnatal mouse brain. **(B)** Validation of the Anti-DGCR8 antibody on chick embryonic brains and postnatal mouse brain by western blot, showing expected 87 and 117 kD bands. The previously observed band at 65 kD has not been identified by the supplier (Abcam).
